# White Matter Infarct Detection with Transformer and Auto-ML-Derived Models

**DOI:** 10.3390/brainsci16050529

**Published:** 2026-05-15

**Authors:** Vitaly Dobromyslin, Wenjin Zhou

**Affiliations:** 1 Francis College of Engineering, University of Massachusetts, Lowell, MA 01854, USA; vitaly_dobromyslin@student.uml.edu; 2 Miner School of Computer & Information Sciences, University of Massachusetts, Lowell, MA 01854, USA

**Keywords:** stroke detection, lesion segmentation, vision transformer, auto-ML, rs-fMRI biomarker, stroke recovery prognosis

## Abstract

Background: The past decade has seen a reversal in the U.S long-term decline in age-adjusted mortality rate from stroke. Timely stroke detection can boost the patient’s chances for recovery by enabling life-saving treatment and informing the patient of their increased risk of successive infarcts. Since no single imaging modality can currently provide accurate and safe stroke detection at both acute and chronic stages, there is a need to develop novel imaging biomarkers with both diagnostic and prognostic value. Methods: We trained a U-shaped, nested hierarchical transformer model (UNesT) for T1-w white matter infarct segmentation using the ATLAS R2 dataset. Model reproducibility was independently evaluated on the Washington University (WU) stroke dataset. To boost T1-w UNesT stroke detection performance, automated machine learning techniques were used to extract 77 novel resting state fMRI (rs-fMRI) stroke biomarkers. Results: Stroke detection performance of the T1-w UNesT model degraded from Dice indices of 0.611 to 0.24 and 0.41 for the subacute and chronic timepoints respectively in the WU dataset. After UNesT re-optimization with the training portion of the WU dataset, the test set Dice index improved to 0.41–0.50. The spectral peak amplitude at the subacute timepoint increased the T1-w UNesT Dice index from 0.41 to 0.50 (*p* < 0.01) and correlated with language recovery. Conclusions: By training a UNesT model on the T1-w stroke data from one dataset and evaluating it on an independent dataset, we highlight the dataset drift concerns. Spectral peak amplitude is proposed as a novel rs-fMRI biomarker for improving stroke detection and predicting stroke recovery trajectory.

## 1. Introduction

Despite decades of continuing declines in the US, age-adjusted mortality rate for stroke is currently seeing a troubling reversal that started in 2014 [1]. This trend, that dis-proportionally affects the younger adults group (25–44 years old), appears to correlate with an upward trajectory in obesity, diabetes, substance use, sedentary lifestyle, and barriers for accessing medical care [2]. According to the U.S. Centers for Disease Control and Prevention (CDC), stroke is the fourth leading cause of death in the US [3]. Some acute (<24 h post-onset) and subacute (1–3 weeks post-onset) stroke episodes may go unnoticed by the patient and a healthcare provider, yet they can cause permanent infarct tissue in the oxygen-deprived areas of the brain, known as chronic (>3 weeks post-onset) infarct. The repeat infarcts are associated with higher risk of successive infarcts and increased morbidity [4].

T1-weighted (T1-w) MRI is the most widely used brain imaging modality; however, it lacks sufficient contrast for reliable stroke detection at acute and subacute phases. Modern approaches to stroke detection aim to automate the time-consuming task of manually reviewing MRI by leveraging deep-learning (DL) frameworks such as convolutional neural networks and vision transformer models. These models are trained for brain tissue segmentation using ground truth lesion masks created by radiologists and strive to achieve the highest possible Dice index (see Equation (1)), a metric ranging from 0 to 1 that balances true positives (TP), false positives (FP), and false negatives (FN).

(1) $$Dice\;Index=\frac{2*TP}{2*TP+FP+FN}$$

Winzeck et al. surveyed multiple acute and subacute stroke T1-w detection studies and reported a Dice index in the 0.05–0.32 range and relatively low inter-rater agreement showing a Dice index of 0.58 ± 0.20 [5]. Diffusion-weighted imaging (DWI) can better detect early signs of stroke in the brain, with a reported Dice index of 0.69 on an independent test set [6]. Over time, the DWI signal stroke signal tends to normalize, making DWI less suitable for subacute and chronic stroke [7]. In cases with suspected acute stroke, CT angiography and CT perfusion provide the gold standard imaging for brain vasculature and salvageable brain tissue respectively. These imaging modalities rely on an invasive contrast agent injection procedure, which has been reported to cause long-term contrast agent deposition in the brain [8]. Resting state fMRI (rs-fMRI) provides a non-invasive alternative imaging technique that probes the blood oxygen level-dependent signal (BOLD) in the brain during rest and can potentially help uncover novel stroke biomarkers. In the past, rs-fMRI has contributed greatly to mapping functional brain connectivity (FC), as well as pathology-related local changes in neuronal metabolism and perfusion.

Stroke can impact both white matter (WM) and gray matter (GM), with damage to the GM typically causing sudden and severe information processing deficits (e.g., specific motor and sensory functions), whereas WM damage is associated with more subtle symptoms such as progressive balance problems and gradual cognitive decline. Historically GM has enjoyed greater research focus due to its involvement in higher order brain functions, compared to the traditional view of WM as merely supporting structural connectivity and maintenance. High synaptic activity within GM drives higher cerebral blood flow (CBF) compared to WM, where CBF level is primarily determined by metabolism supporting signal conduction. The already low CBF in WM makes it particularly vulnerable to any ischemia-related CBF interruption [9]. Overall, WM injury from stroke has been reported to cause as much, and in some cases more, long-term disability compared to GM stroke [10].

The surviving cells in the area surrounding the infarct, referred to as penumbra, often reorganize and rewire themselves to redistribute the lost processing and conduction capacity [11]. With stroke rehabilitation therapy (e.g., physical and language therapy, robot-assisted rehab), this neuroplasticity within penumbra allows for partial patient recovery. Previous rs-fMRI reports include compensatory mechanisms contributing to higher ipsilateral and lower contralateral FC post-stroke [12], region-specific changes in BOLD standard deviation [13], decrease in regional homogeneity (ReHo) [14], and a decreased relative amplitude of low frequency fluctuations (ALFF) [15]. Most up-to-date stroke fMRI studies focus on exploratory whole brain correlational analysis, aimed at revealing regions affected by stroke. While still informative as a data exploration tool, the correlational whole brain analysis does not yield immediately useful clinical information. For example, multiple brain regions may meet the correlation criteria purely as a consequence of running multiple comparisons with a non-negligible false discovery rate. Unlike the Dice index, which explicitly penalizes FPs and FNs, whole brain correlational analysis lacks such “penalty”, making it susceptible to inflating the analysis results. To the best of our knowledge, this is the first study to propose challenging fMRI-derived stroke biomarkers by evaluating Dice index improvement over the baseline T1-w DL model. Our approach expands on the previously reported stroke rs-fMRI biomarkers by extensively screening 77 novel stroke rs-fMRI biomarkers extracted with automated machine learning (Auto-ML) techniques. While most of the 77 Auto-ML features do not have an obvious biological interpretation, many of them come from information theory, signal processing, and statistical analysis, making them well-suited for understanding signal conduction and processing alteration caused by stroke. We further examine rs-fMRI biomarkers’ clinical significance by correlating their levels at subacute and chronic timepoints to motor and neuropsychological assessment results.

## 2. Materials and Methods

### 2.1. T1-w Imaging Data for Chronic Stroke Detection

We used the ATLAS R2 dataset [16] as the starting point for training an infarct detection DL model with T1-w images—see Table 1. Only the training portion (*n* = 655) of the ATLAS R2 dataset was used for this purpose due to the lack of manual lesion segmentation in the test subset. The provided data included a native space T1-w head scan and the accompanying manual lesion segmentation. To perform skull stripping we used a brain extraction tool (BET) [17], which is part of the v6.0.5.2 FSL image analysis suite. Following skull stripping, the native space brain and lesion mask were linearly registered to the MNI152 1 mm template using the FSL FLIRT tool [18].

### 2.2. T1-w Data Augmentation with the Diffusion Model

To increase the training set size for the T1-w DL model we adopted the medical denoising diffusion probabilistic model (MED-DDPM) architecture proposed by Dorjsembe et al. [19]. This model was originally developed for conditional 3D T1-w MRI image synthesis and trained using 1292 tumor patients from National Taiwan University Hospital. The conditional part of the MED-DDPM takes the desired binary anomaly (e.g., tumor) image mask as input and generates a realistic anomaly image using the previously trained diffusion model. For transfer learning from the tumor segmentation to the infarct segmentation domain, we re-trained the original model using 85% (*n* = 557) of the ATLAS R2 dataset, whereas the remaining 15% (*n* = 98) was used for model testing. To comply with the MED-DDPM architecture, native space brain images and lesion masks were linearly registered to the MNI152 1 mm template space and resampled to the 1.5 × 1.5 × 1.5 mm voxel resolution. The T1-w brain images were normalized to have −1 to 1 signal intensity. Stroke masks were modified to have 3 classes: 0 for background, 1 for overall brain area, and 2 for lesion area. We used the default model training parameters in the MED-DDPM original publication [19] (L1 loss, the cosine noise schedule for 250 steps, a learning rate of 10^−5^ for the first 50,000 iterations, and 10^−6^ for the remaining 50,000 iterations), resulting in 179 training epochs.

Considering that DL models typically struggle with detecting small lesions, we focused on augmenting small–medium target lesion masks in the training set (*n* = 24), applying ~4x shrinking, and mirroring to the contra-lateral hemisphere, resulting in 4 mask types (i.e., original, original mirrored, shrunk, and shrunk-mirrored) and 96 total lesion masks.

### 2.3. Hierarchical Transformer Model for Stroke Detection

In this work we adapted a hierarchical hybrid 3D U-shape medical segmentation model with nested transformers (UNesT) [20] as a baseline T1-w lesion detection model. The model was accessed and re-trained through the Medical Open Network for Artificial Intelligence (MONAI) library [21]. While originally the UNesT was developed for segmenting the T1-w brain image into 133 anatomical regions, we re-purposed the model for a binary (i.e., healthy vs. infarct tissue) lesion detection task. The original training set (*n* = 557) data were combined with the synthetic images (*n* = 296) described above to form the augmented training set (*n* = 853). Following the default settings, during the model training the MNI 152 1 mm template-registered images were randomly cropped to size 96 × 96 × 96 voxels, augmented with random flips (with 10% probability), 90° rotations (with 10% probability), and normalized by subtracting the mean and dividing the result by standard deviation.

### 2.4. Calculating Auto-ML Features from rs-fMRI

We used brain imaging data collected at Washington University (WU) School of Medicine, St. Louis [22] to study rs-fMRI biomarkers of stroke. All scans were performed on Siemens 3 T Tim-Trio scanner (Siemens Healthineers, Erlangen, Germany), including sagittal T1-w imaging (TR = 1950 msec, TE = 2.26 msec, flip angle = 9 deg, voxel size = 1.0 × 1.0 × 1.0 mm), rs-fMRI (TR = 2000 msec, voxel size = 4.0 × 4.0 × 4.0 mm, frames = 128), and structural T2-w imaging (TR = 2500 msec, TE = 435 msec, voxel-size = 1.0 × 1.0 × 1.0 mm). Original neurologist-created lesion masks were transformed from the 711-2B Talairach space to the MNI152 space with the provided affine transform. In cases with minor lesion mask misalignment, FSL Nudge tool within FSLeyes [23] was used to align the lesion mask with the respective T1-w and T2-w lesion contour.

Cortical reconstruction and WM segmentation were performed on the native space T1-w images with v7.3.2. FreeSurfer image analysis suite [24]. There were 7 rs-fMRI scans acquired at both subacute and chronic phases. Each rs-fMRI scan underwent discarding of the first 5 frames to allow for scanner stabilization, slice-timing correction [25], motion correction with FSL MCFLIRT tool [26], skull stripping with FSL BET tool [17], and linear registration to the native space T1-w brain image with FSL FLIRT tool [18]. The native space T1-w brain image was linearly registered to the MNI space with the FSL FLIRT tool [18]. The inverse of this transform was used for transforming the MNI space lesion mask to the native subject space.

To enable rs-fMRI stroke biomarker calculation, we added native space lesion and WM masks, followed by 2x spatial down-sampling, resulting in a 2.0 × 2.0 × 2.0 mm voxel resolution region of interest (ROI). Local WM and lesion rs-fMRI signal properties were probed with the 3 × 3 × 3 voxel sliding window, with a minimum cluster size requirement of 2 voxels. Matlab R2023a Predictive Maintenance toolbox [27] functionality was used to extract 77 Auto-ML processing metrics (see Table 1) from the rs-fMRI timeseries in the combined WM and lesion ROI described above. Eight more rs-fMRI features from the literature [28] were also calculated including: low-, mid-, and high-frequency amplitudes of fluctuation; low-, mid-, and high-frequency power ratios; and Kendall Tau and W coefficients. The frequency cutoffs were as follows: 0.01–0.04 Hz for the low-frequency band, 0.04–0.08 Hz for the mid-frequency band, and 0.08–0.10 Hz for the high-frequency band. Due to the limited cohort size, we used a random 50/50% subject-level split at both subacute and chronic time points to preserve enough data in both the training and testing sets while avoiding leakage across repeated rs-fMRI scans from the same subject. The train/test split was performed on the subject level to prevent the repeated rs-fMRI scans for the same subject from being used for both training and testing the model (i.e., data contamination). To test the stability of the observed effects we swapped the original training/testing data split and used the original training data for testing and vice versa. The test set Dice index from the two alternative data splits was averaged and used for final reporting. A modified version of the Fisher score was used to rank the 85 rs-fMRI features in the training set in the descending order of lesion-to-white-matter contrast, see Equation (2).

(2) $${Score}_{i}=\frac{{\left({Mean}_{Lesion}-{Mean}_{WM}\right)}^{2}}{{{SD}_{WM}}^{2}}$$

### 2.5. Combining UNesT Lesion Segmentation with Auto-ML Features

The original T1-w UNesT transformer model’s output consisted of pixel-wise binary values denoting the most likely class as either lesion (class = 1) or non-lesion (class = 0). For the purpose of combining UNesT predictions with rs-fMRI-derived metrics, we disabled the UNesT probability conversion to discrete class and masked out non-WM voxels. Similarly, non-WM voxels were masked out in the rs-fMRI-derived metrics. To standardize data from different scales on individual subject level and to enable better model convergence, we converted raw values to z-scores by subtracting the mean and dividing by standard deviation. At each ROI voxel, infarct log odds (*p*) were modeled with multivariate logistic regression as a general linear model (GLM) including UNesT probability and the best performing rs-fMRI-derived features as inputs—see Equation (3). To limit the potential of rs-fMRI features driving the infarct log-odds in the opposite direction than desired based on the modified Fisher score, depending on the specific case, we zeroed rs-fMRI values with z < 0.1 or −0.1 < z.

(3) $$ln(\frac{p}{1-p})=\beta_{0}+\beta_{1}{*UNesT}_{prob}+\beta_{2}*{fMRI}_{1}+\ldots {\;\beta }_{n}*{fMRI}_{n}$$

Due to the non-convex nature of finding coefficients in Equation (3), multicollinearity, and high number of data points in the 3D ROI image, factor weights were estimated by performing a coarse full-grid search. Other attempted optimization techniques included Nelder–Mead, Powell, and L-BFGS-B algorithms, but they did not yield satisfactory results. These attempts informed the process of establishing the allowed parameter ranges (i.e., grid) for full-grid search. To limit the computation time, only the two strongest rs-fMRI features were modeled. The full grid included 432 unique combinations with 3 levels for offset, 4 levels for UNesT_prob_, 6 levels for the strongest feature, and 6 levels for the second strongest feature. The best model was selected based on the highest mean Dice score on the training subset. When evaluating the GLM on the test set, voxel-wise lesion probability (*p_i_*) was calculated using Equation (4) below. We applied the default p_i_ > 0.5 threshold to transform the final probability map into binary stroke segmentation map.

(4) $$p_{i}=\frac{1}{1\;+\;e^{-(\beta_{0}\;+\;\beta_{1}\;*\;{UNesT}_{prob}\;+\;\beta_{2\;}*\;{fMRI}_{1}\;+\ldots \beta_{n\;}*\;{fMRI}_{n})}}$$

The stroke detection model comparison included three contenders: (1) the original T1-w UNesT model with the default 0.5 binarization threshold, (2) re-optimized T1-w UNesT model threshold using WU training set, and (3) multivariate model featuring T1-w UNesT and the most relevant rs-fMRI biomarker(s), estimated using the WU training set.

### 2.6. Correlation with Motor and Neuropsychological Assessment

At both subacute and chronic visits WU stroke patients’ evaluations included motor skills, word comprehension, and attention assessment. The Functional Independent Measure (FIM) Walk test [29] involved two locomotion items, where the individual’s ability to independently climb stairs and walk on a flat surface was ranked from 1 (completely dependent) to 7 (completely independent). Word comprehension raw scores, part of the Boston Diagnostic Aphasia Exam [30], were used to evaluate patients’ abilities to match spoken words to pictures, objects, and body parts. One point was assigned for each correct answer in the word comprehension task. The Posner Cueing task [31] captured patients’ response times in ms for manual and eye movement to target stimulus, with smaller values indicating faster reaction speed. We performed two types of correlation analyses between rs-fMRI biomarker level and skills assessment scores: (1) prognostic analysis correlating rs-fMRI biomarker level at the subacute timepoint with longitudinal change in the skills assessment, and (2) correlational analysis between longitudinal changes in the rs-fMRI biomarker and skills assessment. Statistical significance was evaluated for each of the two analysis types above based on the 0.05 acceptable family-wise error rate (FWER), controlled with the Holm–Bonferroni method.

## 3. Results

### 3.1. Participant Characteristics

A total of 654 ATLAS R2 and 20 WU research participants’ data were included in the analysis—see Table 2 for additional information. Fourteen of the 20 subacute infarct cohort WU research participants had 1 year of follow-up imaging data available. While the detailed demographic data was not released with the ATLAS R2 dataset, in the WU cohort most infarcts were ischemic, with an average participant age of 52.9 ± 11.5 and 54.6 ± 8.3 for the subacute and chronic infarct cohorts respectively.

### 3.2. Data Augmentation with the Diffusion Model

**Figure 1:**
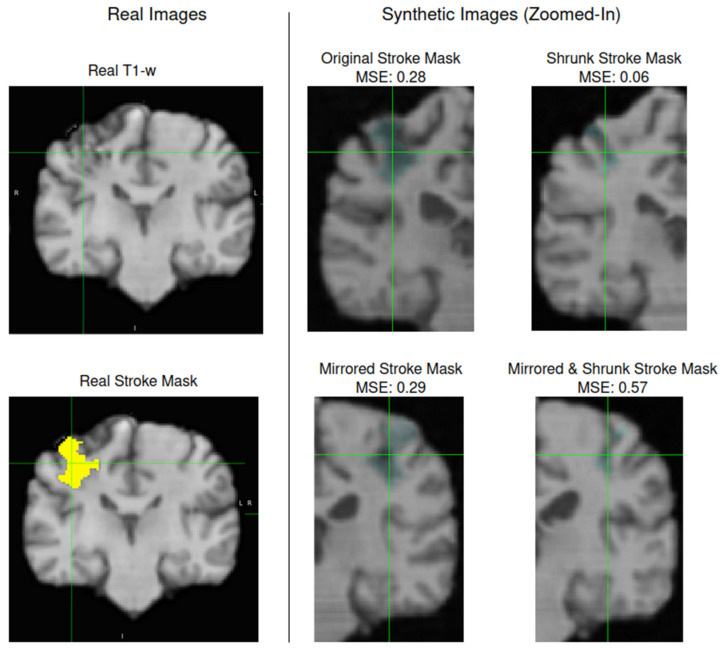
T1-w data augmentation with the MED-DDPM model. Real T1-w (**top left**) and radiologist-created lesion mask (yellow, **bottom left**) images were used for training the MED-DDPM. The diffusion model generated a synthetic T1-w image (grayscale) for the corresponding lesion mask (light blue). We used MSE to assess the generated image fidelity with respect to the original T1-w image.

Taking the input segmentation mask (*n* = 24 unique small and medium lesion cases) with background, overall brain, and lesion segmentation, MED-DDPM generated four synthetic image types—see Figure 1. For each of the four image types, 10 synthetic samples were produced, resulting in 24 × 4 × 10 = 960 generated image pairs (T1-w and segmentation mask). Mean squared error (MSE) calculated between the original and generated T1-w images was used as a criterion (<0.20) to screen out poorly generated images, resulting in a reduction from 960 to 296 generated images. This criterion was experimentally derived by observing the amount of image distortion (e.g., ventricle size, cortical folding pattern, blurring) and clarity of the gray/white matter boundary in the generated images. Two UNesT models were trained: one with original training data and the second model with the augmented dataset from the diffusion model. The latter showed stronger performance, with the ATLAS R2 dataset mean Dice index improving from 0.594 to 0.611, and it was selected as the final UNesT model.

### 3.3. T1-w Infarct Detection with the Transformer Model

**Figure 2:**
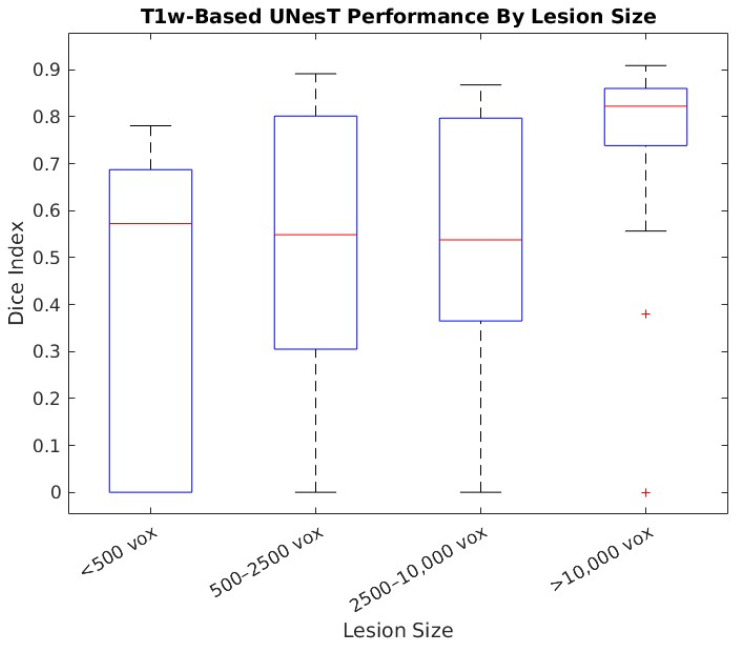
ATLAS R2 test set performance variation by lesion size. UNesT model was used for lesion detection. Samples outside of the median ±1.5xIQR are shown with the “+” symbol.

The T1-w UNesT infarct detection was separately evaluated on the ATLAS R2 test set, WU subacute, and WU chronic infarct cohorts, achieving 0.611, 0.244 and 0.407 mean Dice indices respectively. To characterize UNesT infarct detection performance by lesion size, the ATLAS R2 test set lesions and their respective Dice indices were grouped in four size bins: small (<500 voxels), medium (500–2500 voxels), large (2500–10,000 voxels), and extra-large (>10,000 voxels). As Figure 2 shows, small lesions were associated with a worse detection performance, varying widely in terms of the Dice index compared to the larger lesions.

To test the UNesT reproducibility for detecting subacute infarcts, the UNesT was applied with its original infarct detection criteria of probability > 0.5 to the WU stroke dataset. Compared to the ATLAR R2 test set where the model achieved a 0.61 test set Dice index, in the WU subacute infarct test cohort UNesT performance only reached a 0.24 Dice index—see Table 3. When the UNesT was re-optimized with WU subacute infarct training data (*n* = 10) and univariate (i.e., only using T1-w scans) logistic regression model in Equation (3), the test set average Dice index showed a statistically significant (*p* < 0.01 in the paired *t*-test) improvement from 0.24 to 0.41.

### 3.4. Selecting Subacute Infarct-Specific rs-fMRI Metrics

**Figure 3:**
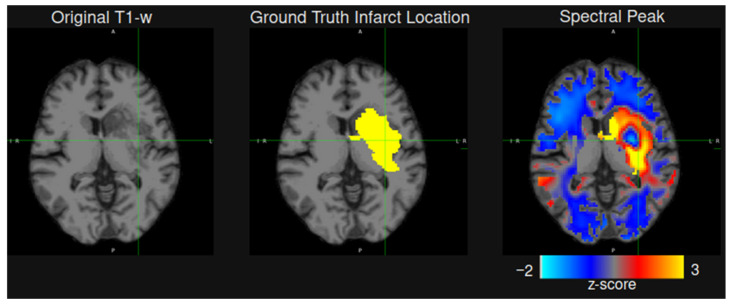
An axial view of subacute infarct on a T1-w MRI (**left**), with the radiologist-created lesion mask (**middle**). The z-transformed image of the spectral peak (**right**) shows encouraging performance for enhancing the lesion contrast. The spectral peak image shown reflects all preprocessing and MNI space transformation.

We evaluated 85 rs-fMRI potential stroke biomarkers in WM based on the signal level in the lesion area compared to the entire WM segmentation mask (i.e., Fisher score)—see Equation (2). The Fisher score was calculated independently for each of the seven rs-fMRI scans collected per patient visit. At the subacute timepoint, spectral peak amplitude showed the highest Fisher score of 1.43 and 2.01 for the original and swapped train/test data splits respectively. In some cases where the lesion had poor contrast on a T1-w scan, spectral peak amplitude offered an exceptionally high contrast, following the lesion contour with high fidelity—see Figure 3. Combining rs-fMRI spectral peak amplitude with UNesT prediction in a single model with Equation (3) yielded a statistically significant (*p* < 0.01 in the paired *t*-test) performance improvement with an average Dice index of 0.50 compared to 0.41 for the T1-w based UNesT model—see Table 3.

### 3.5. Selecting Chronic Infarct-Specific rs-fMRI Metrics

Applying the original UNesT model to the WU chronic infarct cohort yielded a 0.42 ± 0.24 Dice index—see Table 4. After performing UNesT detection threshold optimization with Equation (3) and using 50% of the WU chronic infarct data (*n* = 7 patients, 49 rs-fMRI scans) as training data, the mean test Dice index improved from 0.42 to 0.50. The methodology for identifying promising chronic infarct rs-fMRI biomarkers closely followed the approach described for subacute infarcts above. Out of the 85 potential rs-fMRI biomarkers, autoregression Akaike Information Criteria (AIC) had the highest average Fisher score of 0.37, followed closely by a 0.34 Fisher score for the spectral peak amplitude. Autoregression AIC reflects how well the past values in the rs-fMRI timeseries predict the current value, with lower AIC values indicating better fit with respect to model complexity. When both autoregression AIC and spectral peak amplitude were used in Equation (3) as part of the stroke detection model optimization, the autoregression AIC factor became zeroed out in favor of the spectral peak amplitude. Combining spectral peak amplitude with UNesT model did not yield a statistically significant improvement in terms of the test set Dice index.

**Figure 4:**
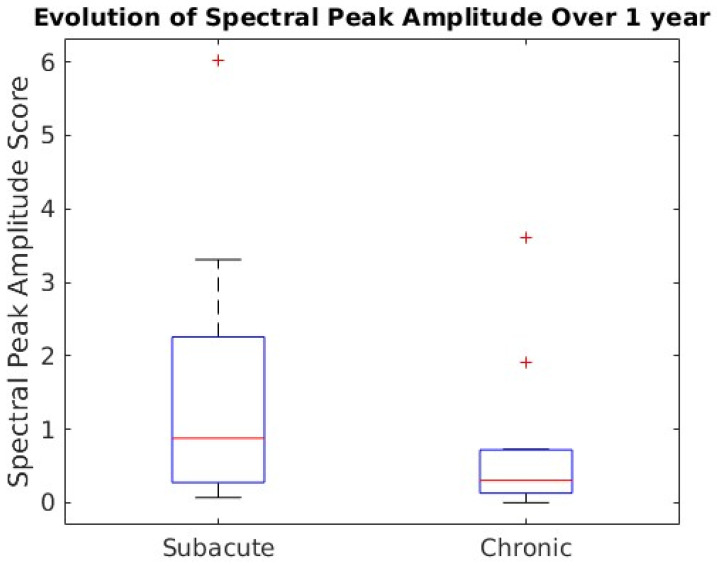
Average Fisher scores for spectral peak amplitude at subacute and chronic timepoints. The decline from subacute to chronic phase was statistically significant, with *p* = 0.029 in the paired *t*-test. Samples outside of the median ±1.5xIQR are shown with the “+” symbol.

Overall, the mean spectral peak amplitude showed a statistically significant decline from 1.43 to 0.66 between subacute and chronic visits, with *p* = 0.029 in the paired *t*-test—see Figure 4.

### 3.6. rs-fMRI Association with Motor and Neuropsychological Assessment

Stroke recovery was assessed with the change in motor skills, word comprehension and attention shift task scores between chronic and subacute visits over the course of roughly 1 year, see Table 5. The first family of statistical tests consisted of evaluating the association between subacute timepoint spectral peak amplitude and the assessment scores above. The Holm–Bonferroni test yielded the corresponding step-wise *p*-value thresholds of {0.017, 0.025, 0.05}, with only the change in word comprehension meeting the statistical significance criteria.

The second family of statistical tests consisted of evaluating the association between changes in subacute timepoint spectral peak amplitude and assessment scores. None of the effects in the second family of statistical tests met the Holm–Bonferroni adjusted statistical significance criteria.

**Figure 5:**
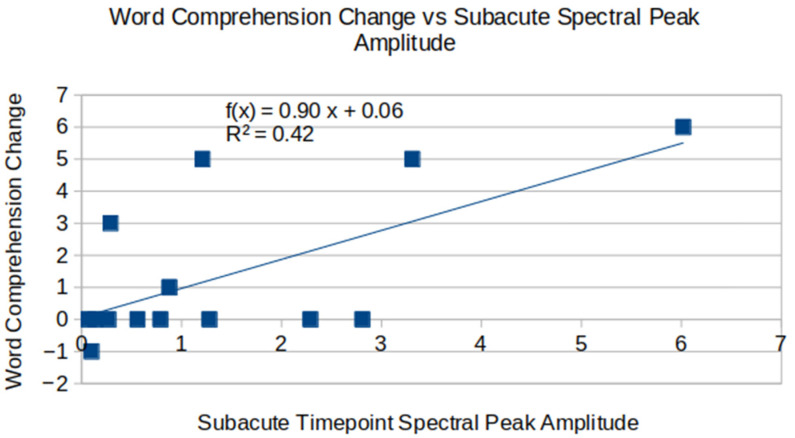
Spectral peak amplitude at the subacute stroke phase appears to show prognostic value for improvement in word comprehension over the course of 1 year. The plot includes data from 14 patients with the available subacute and chronic visit assessment scores.

Spectral peak amplitude during subacute visits showed prognostic value for the change in word comprehension (*p* = 0.012, R^2^ = 0.42), with higher spectral peak amplitude being associated with an improvement in word comprehension ability—see Figure 5.

**Figure 6:**
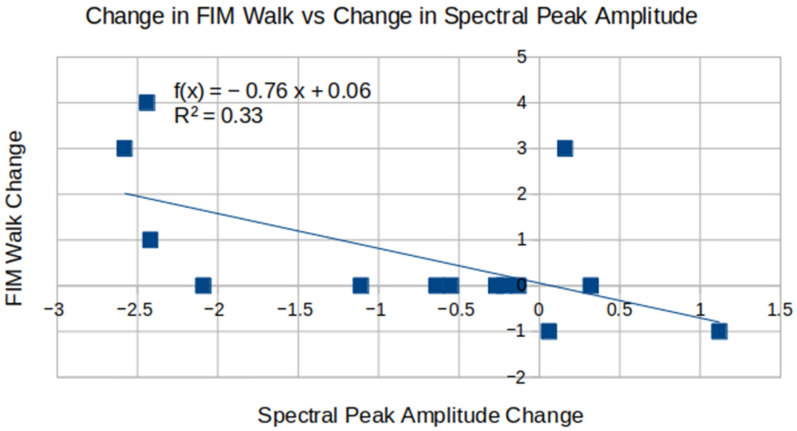
Improvement in FIM Walk score inverse relationship with the change in spectral peak amplitude. The plot includes data from 14 patients with the available subacute and chronic visit assessment scores.

The change in FIM Walk score showed inverse proportionality to the spectral peak amplitude change between the subacute and chronic visit, with *p* = 0.031 and R^2^ = 0.33—see Figure 6.

## 4. Discussion

DL is enjoying a growing research interest due to its potential for improving disease detection and alleviating some healthcare system resource bottlenecks. In this study we adapted a state-of-the-art vision transformer UNesT model for stroke detection with T1-w MRI data. Our experiments showed that while the vision transformer model achieved an encouraging (0.611 Dice index) performance on one multi-institution dataset, its performance noticeably degraded (0.24–0.41 Dice index) when applied to the independent test set. The reproducibility drop in this study echoes the earlier report by Gryska et al. examining reproducibility of the published DL brain tumor segmentation models on an independent dataset, and in some cases observing the Dice index dropping from 0.78 to 0.36 [32]. A number of factors likely contribute to the DL model performance variation, including: scanner-related differences (e.g., 1.5 T vs. 3 T magnetic field, manufacturer-to-manufacturer differences), lack of imaging sequence standardization, and differences in patient demographics. Yan et al. examined MRI manufacturer impact on the trained CNN model performance for a segmentation task and observed 23.4–34.3% drop in the Dice index when the model was trained and tested on MRI systems from different manufacturers [33]. In terms of the magnetic field strength, Puzio et al. found more consistent DL-based brain structure segmentation on 1.5 T compared to the 3 T MRI scanners [34]. Even on the same MRI scanner, DL-based brain structure segmentation has been observed to produce 7–8% volume variation for the same research participant [35]. Our study provides further evidence that model reproducibility, and in some cases model re-optimization, need to be addressed before widespread model deployment.

By systematically screening 77 novel Auto-ML and eight previously reported (e.g., ALFF, fALFF, ReHo, etc.) rs-fMRI features with a modified Fisher score, we were able to identify spectral peak amplitude as the strongest rs-fMRI biomarker for improving the baseline T1-w UNesT model performance. The combined multivariate model showed a statistically significant improvement in subacute stroke detection over the re-trained T1-w UNesT model, with the corresponding Dice index improving from 0.41 to 0.50. In comparison to ALFF and fALFF metrics, where the frequency cutoffs are hard-coded to be the same across all subjects, the spectral peak amplitude is data-driven by the actual peak in the subject’s rs-fMRI spectrum. This data-driven nature of the spectral peak amplitude may boost its robustness in the presence of subject-to-subject differences. One way to interpret our spectral peak amplitude observations in the time domain involves viewing it as a manifestation of a strong periodic signal, serving a potentially protective purpose. Russo et al. used EEG to examine the impact from controlled lesions in patients undergoing ablation therapy and reported a perilesional rise in sleep-like slow-wave activity [36]. Cassidy et al. reported resting-state EEG delta (1–3 Hz) band power correlation with 1) stroke volume and 2) better recovery status in the chronic infarct stage [37]. In the mouse stroke model, Facchin et al. demonstrated the beneficial effect of selectively inducing sleep-like slow-wave activity for improving fine motor movements of the limb corresponding to the sensorimotor stroke lesion site [38]. While the rs-fMRI repetition time of 2 s in the current study limits the temporal resolution to an upper (Nyquist) frequency of 0.25 Hz, the elevated spectral peak amplitude in the perilesional area observed in our study may reflect aliased components of the sleep-like slow waves reported in the abovementioned EEG studies. At this stage, the comparison between spectral peak amplitude and sleep-like slow waves requires additional investigation and therefore is hypothetical in nature.

This study showed prognostic value from WM rs-fMRI spectral peak amplitude for explaining 42% of word comprehension improvement after 1 year post-stroke onset. Higher spectral peak amplitude at the subacute timepoint was associated with greater improvement in word comprehension. Unlike other approaches aimed at tracking long-range FC disruptions (e.g., seed-based connectivity, ICA, signal delay studies), our study considered local rs-fMRI pattern probed with a 6 × 6 × 6 mm sliding window, greatly simplifying the analysis workflow. In a related long-range FC connectivity study, Fruhwirth et al. reported 15.3% of the processing speed improvement 3 months post-stroke explained by the anterior cingulate cortex FC [39]. Using DWI to probe WM integrity association with neuropsychological assessment scores, Schellekens et al. reported that 30–37% of the processing speed variance over a 6-month period was explained by fractional anisotropy values in the affected hemisphere [40]. With our approach, targeted specifically at probing local WM functional changes, we achieved a higher stroke recovery prognostic value than previous reports. Despite the encouraging preliminary results showing spectral peak amplitude as a promising subacute stroke biomarker, further validation in prospectively collected, multi-site cohorts is needed.

Several limitations exist in this study, including limited sample size, lack of data at the acute stroke timepoint, and lack of the age-matched control cohort for evaluating rs-fMRI biomarkers. Future studies will benefit from combining brain imaging with physiological monitoring data to help decouple potential contribution from breathing and blood circulation.

## 5. Conclusions

Our findings show that WM stroke detection using T1-w imaging remains a challenging task for the existing DL-based approaches. Scanner-to-scanner differences, non-harmonized image acquisition parameters, varying patient demographics, and varying time post-stroke onset are likely contributors to the performance differences we saw between the two independent datasets included in this study. At the subacute stroke phase, rs-fMRI-derived spectral peak amplitude appears as a promising candidate biomarker, with a potential to improve T1-w diagnostic performance and offer prognostic insights related to patient recovery. More studies are needed to clinically validate this biomarker for infarct detection and recovery prediction.

## Figures and Tables

**Table 1:** Auto-calculated metrics by Matlab Diagnostic Feature Designer app.

*Raw Signal Features*	Q1	*Power Spectrum Features*	Power	Maximum
Clearance Factor	Q3	Peak Amplitude	*Linearly De-trended Features*	Q1
Crest Factor	IQR	Peak Frequency	Clearance Factor	Q3
Impulse Factor	*Signal Envelope Features*	Power	Crest Factor	IQR
Kurtosis	Clearance Factor	*Features After Autoregressive Filtering*	Impulse Factor	Shape Factor
Mean	Crest Factor	First Coefficient	Kurtosis	*Linearly De-trended, After Autoregressive Filter, Features*
Peak Value	Impulse Factor	First Frequency	Mean	First Coefficient
RMS	Kurtosis	MSE	Peak Value	First Frequency
SINAD	Mean	MAE	RMS	Damping Coefficient
SNR	Peak Value	AIC	SINAD	MSE
Shape Factor	RMS	Mean	SNR	MAE
Skewness	SINAD	Variance	Shape Factor	AIC
SD	SNR	RMS	Skewness	Mean
THD	Shape Factor	Kurtosis	SD	Variance
Minimum	Skewness	*Signal Envelope Spectral Features*	THD	RMS
Median	SD	Peak Amplitude	Minimum	Kurtosis
Maximum	THD	Peak Frequency	Median	

**Table 2:** Population demographics for each of the two datasets used.

Dataset	*n*	Median Days Post-Stroke	Median Lesion Volume [mm^3^]	Infarct Type Count	Age Mean ± SD	Gender % Male % Female
ATLAS R2 Subacute–Chronic Cohort	654	501	3996	*	*	*
WU Subacute Infarct Cohort	20	12	37,914	16 ischemic 1 hemorrhagic 3 other	52.9 ± 11.5	40.0% M 60.0% F
WU Chronic Infarct Cohort	14	382	36,235	11 ischemic 1 hemorrhagic 2 other	54.6 ± 8.3	22.2% M 77.8% F

* No data provided with the release.

**Table 3:** Subacute infarct Dice index in the test set using UNesT and a multivariate model. Where applicable, standard deviation is included next to the mean. The multivariate model showed a statistically significant improvement compared to the re-optimized UNesT model with *p* < 0.01 in the paired *t*-test.

Model	Data Split	Subj. 1	Subj. 2	Subj. 3	Subj. 4	Subj. 5	Subj. 6	Subj. 7	Subj. 8	Subj. 9	Subj. 10	Mean ±SD
Original UNesT *	Original Train/Test	0.00	0.55	0.00	0.67	0.02	0.08	0.00	0.50	0.07	0.54	0.24 ±0.28
Re-Optimized UNesT *		0.03	0.68	0.11	0.74	0.10	0.37	0.00	0.77	0.45	0.83	0.41 ±0.32
Multivariate GLM		0.10 ±0.00	0.64 ±0.00	0.39 ±0.01	0.78 ±0.00	0.65 ±0.01	0.42 ±0.00	0.00 ±0.00	0.80 ±0.00	0.52 ±0.01	0.85 ±0.00	0.51 ±0.28
Original UNesT *	Swapped Train/Test	0.11	0.49	0.66	0.01	0.57	0.00	0.00	0.01	0.47	0.63	0.29 ±0.29
Re-Optimized UNesT *		0.56	0.57	0.58	0.08	0.42	0.00	0.37	0.09	0.85	0.44	0.40 ±0.26
Multivariate GLM		0.69 ±0.01	0.51 ±0.01	0.43 ±0.00	0.33 ±0.01	0.15 ±0.06	0.00 ±0.00	0.62 ±0.01	0.75 ±0.01	0.89 ±0.00	0.36 ±0.00	0.49 ±0.26

* The WU dataset included a single structural T1-w scan, making SD calculation impractical.

**Table 4:** Chronic infarct Dice index in the test set using UNesT and a multivariate model. Where applicable, standard deviation is included next to the mean.

Model	Data Split	Subj. 1	Subj. 2	Subj. 3	Subj. 4	Subj. 5	Subj. 6	Subj. 7	Mean ±SD
UNesT *	Original Train/Test	0.58	0.30	0.01	0.45	0.85	0.53	0.14	0.41 ±0.28
Re-Optimized UNesT *		0.67	0.44	0.06	0.53	0.86	0.51	0.33	0.49 ±0.25
Multivariate GLM		0.67 ±0.00	0.43 ±0.01	0.06 ±0.00	0.54 ±0.00	0.86 ±0.00	0.51 ±0.00	0.37 ±0.00	0.48 ±0.23
UNesT *	Swapped Train/Test	0.53	0.63	0.00	0.47	0.47	0.43	0.43	0.42 ±0.20
Re-Optimized UNesT *		0.65	0.55	0.00	0.43	0.72	0.59	0.58	0.50 ±0.24
Multivariate GLM		0.66 ±0.00	0.47 ±0.00	0.00 ±0.00	0.37 ±0.01	0.72 ±0.00	0.69 ±0.00	0.61 ±0.00	0.51 ±0.23

* The WU dataset included a single structural T1-w scan, making SD calculation impractical.

**Table 5:** Correlation between spectral peak amplitude and motor and neuropsychological assessment scores.** Denotes statistically significant results with FWER of 0.05 controlled with the Holm–Bonferroni method.

Independent Variable	Dependent Variable	Slope Estimate	*p*-Value	R^2^
Subacute Timepoint Spectral Peak Amplitude	Change in Word Comprehension	0.90	0.012 **	0.42
Spectral Peak Amplitude Change	Change in FIM Walk	−0.76	0.031	0.33
Spectral Peak Amplitude Change	Change in Word Comprehension	−0.95	0.083	0.23
Subacute Timepoint Spectral Peak Amplitude	Change in FIM Walk	0.41	0.108	0.20
Subacute Timepoint Spectral Peak Amplitude	Change in Posner Cueing	−36.40	0.603	0.04
Spectral Peak Amplitude Change	Change in Posner Cueing	33.74	0.622	0.03

## Data Availability

Data available in a publicly accessible repository. ATLAS R2 (https://fcon_1000.projects.nitrc.org/indi/retro/atlas.html (accessed on 12 May 2026)); Washington University (https://cnda.wustl.edu/app/template/Login.vm (accessed on 12 May 2026); Study ID: CCIR_00299).

## Abbreviations

The following abbreviations are used in this manuscript:

AIC	Akaike Information Criteria
ALFF	Amplitude of low frequency fluctuations
Auto-ML	Automated machine learning
BET	Brain extraction tool
BOLD	Blood oxygen level dependent
CBF	Cerebral blood flow
CDC	U.S. Centers for Disease Control and Prevention
DL	Deep learning
DWI	Diffusion-weighted imaging
FC	Functional connectivity
FIM	Functional independent measure
FN	False negative
FP	False positive
GLM	General linear model
GM	Gray matter
MED-DDPM	Medical denoising diffusion probabilistic model
MONAI	Medical Open Network for Artificial Intelligence
ReHo	Regional homogeneity
ROI	Region of interest
rs-fMRI	Resting-state fMRI
TP	True positive
T1-w	T1-weighted
UNesT	U-shape nested transformers
WM	White matter
